# The Palliative Treatment Plan as a Bone of Contention between Attending Physicians and Nurses

**DOI:** 10.3390/healthcare3040987

**Published:** 2015-10-16

**Authors:** Wolfgang Lederer, Stefanie Graube, Angelika Feichtner, Elisabeth Medicus

**Affiliations:** 1 Department of Anaesthesiology and Critical Care Medicine, Innsbruck Medical University, Innsbruck A-6020, Austria; E-Mail: Stefanie.graube@student.i-med.ac.at; 2 Institute for Palliative Care and Organisational Ethics, Faculty of Interdisciplinary Studies, IFF Vienna Campus and Alpen-Adria University, A-1070 Vienna and Paracelsus Medical University, Salzburg A-5020, Austria; E-Mail: angelika.feichtner@gmx.net; 3 Hospice and Palliative Care, Tyrolean Hospiz-Betriebsges.M.B.H., Innsbruck A-6020, Austria; E-Mail: elisabeth.medicus@hospiz-tirol.at

**Keywords:** palliative care, emergency medicine, geriatric medicine, nursing home, advance care planning

## Abstract

Acute vital crisis in end-of-life situations may result in hospitalization and intensive care without recognizable benefit in many cases. Advance directives regarding indications for resuscitation, hospitalization, and symptomatic treatment help ensure that acute complications can be managed quickly and satisfactorily in the patient’s customary surroundings. A plan was designed and implemented in Austrian nursing homes to provide emergency physicians with rapidly obtainable information on the patient’s current situation, and whether resuscitation attempts and hospitalization are advised or not. This palliative treatment plan is arranged by a physician together with caregivers, close relatives, and the patient or his court-appointed health care guardian or holder of power of attorney. Four years after implementation of the plan, a user satisfaction survey was carried out. The majority of participating nurses, emergency physicians and family doctors judged application and design of the palliative treatment plan positively. However, the low response rate of family doctors indicates nonconformity. In particular, the delegation of symptomatic treatment to nurses proved to be controversial. There is still a need to provide up-to-date information and training for health professionals in order for them to understand advance directives as extended autonomy for patients who have lost their ability to make their own decisions.

## 1. Introduction

The process of planning for future medical care respects a patient’s wishes and general preferences, particularly for situations when the patient becomes unable to participate in decisions about treatment and care [1,2]. Advance care planning for patients in palliative care can help prevent end-of-life crises and ensure high satisfaction of patients, families, and caregivers [3]. Wishes of family members have no legal effect under Austrian law. Directives are binding only when given by court-appointed guardians, but the binding nature of such directives can be enhanced when the patient has already discussed his or her advance care issues with the guardian [4]. In 2006, the legal basis for a living will was established in Austria. Since then, about 5% of the population has made one. Alas, adherence to the copious verbalization of a patient’s living will is not practicable in emergency conditions. Unambiguous agreements and a realistic choice of therapy options proposed by family doctors are vital [5]. This information is particularly important when a life-threatening emergency arises during palliative care, necessitating a call for emergency medical services (EMS).

In order to prevent burdens to patients caused by loss of intimate environments and discontinuation of palliative care, a custom-tailored palliative treatment plan (PTP) was developed and introduced as a kind of check-list in 2010 [6]. Besides advanced symptomatic treatment, the PTP also provides information on whether resuscitation attempts and intensive care are regarded as useful or not by physicians, who have known the patient well for many years. This information is rapidly accessible to nurses and emergency physicians within a few seconds. Four years after implementation of the PTP a prospective assessment aimed to analyze the degree of satisfaction with its application.

## 2. Experimental Section

A prospective study was conducted at the nursing home Kompetenzzentrum Rum (social center for competence), one of 14 nursing homes in Innsbruck County, Austria. This nursing home was the first institution to implement the PTP. At the time of study planning, the nursing personnel had experience with the PTP for more than four years. Eligible participants were nurses and family doctors who currently cared for palliative patients in the nursing home, and emergency physicians from the competent university-affiliated, physician-staffed EMS. Nurses, family doctors, and emergency physicians who voluntarily participated in this survey were enrolled. Information on the study and letters of agreement were distributed or mailed to all eligible participants to ask for written informed consent prior to commencement of the study.

### 2.1. Palliative Treatment Plan

When a patient is admitted to the nursing home, his family doctor continues providing medical care. However, the family doctor may not be readily available. Consequently, in case of acute vital crisis and in end-of-life situations, the nurse on duty calls for the emergency physician. In such comprehensive care settings with limited information on the patient’s situation, the emergency physician has to decide quickly whether the burden arising from further hospitalization or from certain medical treatment options is reasonably balanced by the potential benefits of the steps taken. Therefore, palliative care nurses and physicians from Hospice and Palliative Care, as well as from the university-affiliated emergency medical system (EMS) of Innsbruck City developed a palliative treatment plan as an instrument for advance care planning in 2010 [6]. The PTP can provide immediately accessible information on the patient’s situation and whether hospitalization and/or resuscitation attempts can be viewed as inappropriate or not. Furthermore, the plan prescribes symptomatic treatment that can be delegated to graduated nurses to meet the patient’s immediate needs.

The front page contains the patient’s name and date of birth, the primary diagnosis, and relevant secondary findings. The PTP clearly states whether cardiopulmonary resuscitation (CPR) is currently advised or not, and whether hospitalization is advocated or not when life-threatening emergencies necessitate an EMS call. Information is provided regarding the patient’s ability to make decisions and their legal capacity, availability of a patient’s living will, existence of a court-appointed health care guardian or holder of power of attorney, and on the refusal of medical treatment by presumed patient will. The PTP also clearly states whether relatives were informed about the current situation.

The reverse side of the treatment plan gives instructions for symptomatic treatment in case of pain, dyspnea, agitation, anxiety, confusion, nausea and vomiting, and others if indicated. It displays phone numbers of the family doctor and his substitute, close relatives, holder of power of attorney, and, if requested, spiritual guidance (Supplementary Tables S1 and S2).

### 2.2. Questionnaire

A self-reporting questionnaire was developed for assessment of user satisfaction with the PTP and was submitted to nurses, family doctors, and emergency physicians (Supplementary Table S3). The questionnaire contains 30 questions, 14 of which regard PTP application including patient benefit, depository, reliability, ease of work decisions, ease of use, efficacy, accessibility and design, and 16 questions regarding content including clearness, information quantity, information sequence, transparency, and update status.

Participants gave their responses either on a five-point Likert scale (1 = excellent, 5 = very poor) or in their own words. In addition, suggestions for improvement were requested.

Before commencement of the survey, the questionnaire and the PTP were sent to the administrative head of a nursing home in Germany with the request to check for comprehensibility and applicability of the domain of aspects to be measured. The final version of the questionnaire was modified according to this expert opinion on content validity. From a previous study regarding satisfaction of patients, nurses and physicians with emergency medical care in geriatric nursing homes, we expected most responses and most confirmation of the PTP from nursing personnel [7]. However, the questionnaire was not tested for validity and reliability, as this was the first survey on PTP user satisfaction without repeated testing of the same participants at different times.

Questionnaires were distributed in printed form at the nursing home Kompetenzzentrum Rum in Innsbruck County. Furthermore, questionnaires were distributed by e-mail to family doctors who currently cared for patients in this nursing home, and to emergency physicians from the competent university-affiliated, physician-staffed EMS who were on duty within the last four years.

As questionnaires were completed and returned anonymously, there was no opportunity to re-survey initial non-responders. Participation was voluntary and based on the understanding that results will be published in scientific journals. Procedures followed were in accordance with the Helsinki Declaration of 2004 [8], revised in 2008 [9]. After a complicated appraisal process, the study protocol was finally approved by the Ethics Committee of AN2014-0173.

### 2.3. Statistical Analysis

Demographic data are presented as frequencies and percentages. Comments in the open ended sections were categorized according to topics and discussed in Section 3.3: Strengths and weaknesses of the PTP. Ordinal variables were analyzed with the Mann-Whitney U Test (*n* = 2). Answers given on a five-point Likert scale (1 = excellent, 5 = very poor) are presented in cumulated categories as confirm (1 and 2) or disagree (3, 4 and 5). Correlations between ordinal and/or metric variables were calculated with Spearman Rho. Results were deemed significant for a *p*-value ≤ 0.05.

## 3. Results and Discussion

Four years after PTP implementation in the nursing home Kompetenzzentrum Rum, an assessment was conducted to analyze satisfaction with its application. Responses from nurses, emergency physicians, and family doctors who participated in the survey were mostly positive. It appeared that those who frequently used PTP were those most likely to confirm its usefulness. At study commencement 20 (29.9%) of 69 patients admitted to Kompetenzzentrum Rum were currently treated according to a PTP. Employed at the nursing home were 39 nurses (36 female, 3 male), of whom nine (23.1%) were full-time employees. 19 nurses participated in the study (response rate: 48.7%). Of a total of 50 attending emergency physicians 15 (8 female, 7 male) participated in the study (response rate 30.0%). Twelve attending family doctors were contacted, but only three participated in the study (response rate 25.0%). The majority of nurses (57.9%) had experience with five or more patients being treated according to a PTP. All participating family doctors had experience with the PTP during patient treatment, but only one-fifth of emergency physicians knew of the plan from emergency operations. The results regarding user satisfaction are shown in Table 1.

### 3.1. Questions Regarding PTP Application

Three-quarters of emergency physicians and all but one of the nursing staff underlined the importance of having rapid access to PTP information. All emergency physicians and all but one of the nurses viewed the plan as benefitting the patients and all agreed that PTP provides more reliability in the patients’ medical care. The overall majority of nurses and emergency physicians were satisfied with PTP implementation. The three participating family doctors confirmed that the plan was rapidly accessible and benefitted the patients.

**Table 1:** Satisfaction with palliative treatment plan (PTP) regarding patient benefit, information sequence, clearness, accessibility, reliability, ease of work decisions, ease of use, efficacy and information quantity as reported by nurses and emergency physicians (Confirm is 1 and 2; disagree is 3, 4 and 5 on the Likert Scale).

Characteristics	Nurses (*n* = 19)		Emergency Physicians (*n* = 15)	
	Confirm	Disagree	Confirm	Disagree
patient benefit	18	1	15	0
information sequence	15	4	13	2
clearness	15	4	12	0
accessibility	18	0	11	4
reliability	18	1	14	1
ease of work decisions	16	2	15	0
ease of use	11	5	8	7
efficacy	17	2	13	2
information quantity	14	4	8	7

### 3.2. Questions Regarding PTP Design

All participating family doctors were satisfied with PTP ease of use, outline, and clarity. The majority of emergency physicians and nursing personnel were satisfied with the outline of the PTP and its information content. One-third of nursing personnel, two-thirds of emergency physicians and the three participating family doctors recommended monthly updates.

Nursing personnel who declared they were very satisfied with PTP ease of use also stated that the PTP was very efficient (*p* = 0.044) and high facilitation (*p* = 0.044). High facilitation correlated with assumed patient benefit (*p* = 0.062). In emergency physicians, high satisfaction with PTP ease of use correlated with outline (*p* = 0.000), clarity (*p* = 0.001), facilitation (*p* = 0.001), efficiency (*p* = 0.001) and information content (*p* = 0.001).

### 3.3. Strengths and Weaknesses of the PTP

Emergency physicians stressed that rapid access to detailed information on emergency medical treatment advocated by the family doctor is crucial. Nurses focused more on the importance of predetermined instructions in complication management. Emergency physicians and nurses regarded information on medical treatment that is advocated and that forms the background for decisions to be paramount. Information on the availability of a living will, as well as the patient’s ability to make decisions and his legal capacity, and the fact that relatives were informed was regarded as important. Nurses and emergency physicians requested that persons who participated in advance care planning be named in the PTP, and that a patient’s declared intentions should be given stronger emphasis.

Emergency physicians criticized that the PTP provided no clear distinction between curative and palliative approach and that information on the advanced course of disease needs to be more precise. Nursing personnel requested that legal information be provided for cases when CPR is not to be attempted. Furthermore, information for potential complications should be provided from predetermined arrangements. In symptomatic treatment, localization and intensity of pain should be considered, as well as additional measures including infusion pumps for analgesia.

### 3.4. Limitations and the Potential of Conflicts

The informative value of this survey is limited by the fact that the majority of nurses had experience with five or more patients treated with the assistance of a PTP, but only one-fifth of emergency physicians had experience with a PTP in patient treatment. It is to be assumed that those who mostly disagreed with the PTP did not respond. Furthermore, the questionnaire was not tested for validity and reliability.

Colon *et al.* observed that confusion with clinical practice guidelines, checklists, and regulations was common in nurses working in nursing homes, and that checklists could replace clinical judgment and conflict with facility policies [10]. Ethical problems may arise when the principle of beneficence conflicts with autonomy, justice or loyalty [11]. Withholding intensive care can still be seen as malpractice despite the fact that in an end-of-life crisis supportive treatment is more adequate. At least for the PTP, this checklist seems to have been well accepted by the nursing personnel in our survey.

The complicated appraisal process of the Ethics Committee reflects the ambiguous attitude towards medical care in end-of-life situations. Whereas the committee members announced only negligible comments on the questionnaire, it took several months to come to an agreement on the PTP. This is quite amazing, because the PTP has been legally approved as a checklist to avoid emergency treatment and hospitalization without recognizable benefit to the patient. Furthermore, in the first year of PTP implementation, the plan was introduced in the official journal of the Tyrolean Medical Association as being trend-setting in advance care planning [12].

Emanuel *et al.* reported that of the perceived barriers to issuing advance directives, the lack of physician initiative was among the most frequently mentioned. Many physicians are hesitant and fail to initiate discussions regarding advance planning in palliative care because they fear that patients are uncomfortable discussing issues surrounding their own mortality [13]. On the other hand, it is well known that physicians who initiate conversations on advance care planning can do a lot to diminish patient stress and anxiety [14].

Another conflict seems to arise from competing responsibilities between nursing personnel and family doctors. On the one hand, it is generally accepted nowadays that 24-h availability can no longer be expected of a family doctor. On the other hand, family doctors are reluctant to delegate symptomatic treatment to registered nurses and to clearly determine whether CPR and hospitalization are advised or not should life-threatening emergencies necessitate an EMS call.

In our experience, the situational delegation of symptomatic treatment to nurses, and preparatory training of personnel can diminish EMS calls in nursing homes. One year after PTP implementation, it was noticed that the number of EMS calls had dropped from previously 16 to currently four calls within a year [6]. Presumably, the number of calls without recognizable benefit to the patient had decreased. It was further reported that in some instances PTP implementation created the basis for an intense conversation between family doctors, nurses, and patients about targets of medical care [6].

Problems and conflicts between nurses and doctors in the hospital were reported to stem from the organizational structure of the hospital rather than from the personalities of the individuals involved [15]. In urgent cases, nurses are under pressure to take over responsibility and coordinate patient care without having the requisite authority [15]. In such cases, nurses make decisions and act by contacting other healthcare team members, making referrals and coordinating care with other institutions [16]. Although these actions are for the immediate benefit of patients, they are not a substitute for the development of needed organizational changes in nursing care [15].

Conflicts in communication are predetermined whenever anticipations differ between various occupational groups, relatives and patients [17]. In a previous nursing home study, we observed impaired communication between nursing personnel and emergency physicians [7]. Nurses more frequently found fault with emergency physicians’ style of communication, and complained of their arrogance, haughtiness, and lordliness. On the other hand, emergency physicians more frequently found fault with nurses’ quality of communication and complained of their lacking perspective and information [7]. However, it was not investigated whether communication between family doctors and nursing personnel is also impaired.

## 4. Conclusions

Crisis episodes frequently mark the beginning of the dying process. Without clear directives based on the patient’s will and approved in advance care planning, conflicts with what physicians, nurses, or surrogates view as being in the patient’s best interest are predictable [18]. Most participants in this survey agreed on the importance of advance directives and rapid provision of information. PTP, as an instrument for advance care planning set up by the attending family doctor, together with the caregiver and relative, the patient or his guardian or holder of power of attorney, can help prevent a burden to palliative care patients and their relatives.

## Supplementary Files

Supplementary File 1
