# Supplementary material for: The Palliative Treatment Plan as a Bone of Contention between Attending Physicians and Nurses
Source: Healthcare (Basel). 2015 Oct 16;3(4):987–94. doi: 10.3390/healthcare3040987 (PMC4934626; doi:10.3390/healthcare3040987)
Supplement: Supplementary File 1 [file healthcare-03-00987-s001.pdf]

Supplemental Materials

Table S1. Plan for crisis and emergency management—Palliative Treatment Plan (to be completed by family doctor and attachment nurse).

**Name:**	**Date of birth:**
Medical Background<br>Chief diagnosis and relevant secondary findings<br>Medical improvement is not to be anticipated. The current situation demands predominant palliative treatment	
Cardiopulmonary resuscitation is not advised	☐ Appropriate<br>☐ Not appropriate
Hospitalization is to be avoided if possible	☐ Appropriate<br>☐ Not appropriate
**Background of Decision**	
Ability to decide and to judge is present	☐ Yes<br>☐ Impaired<br>☐ Diminished<br>☐ No
Patient will is on hand	☐ Yes<br>☐ no
Authority to proxy is given	☐ Yes<br>☐ no
Solicitor is announced	☐ Yes<br>☐ no
Refused medical treatment by patient<br>(presumed patient will)	☐ Yes<br>☐ no
**Comments:**	
Relatives are informed about the current situation	☐ Yes<br>☐ no
**Comments:**	

Table S2. Symptomatic Treatment Plan (*Adapted to the individual patient*).

**Indication**	**Medication/Treatment**	**Dose Mode of Application**	**Max. Dose/Day Dose Intervals**
Pain			
Dyspnea			
Anxiety/Agitation			
Nausea/Vomiting			
Rales			
Others: (fever, confusion, seizures, …)			
Main attachment person (name/s, phone number, function)			
Family doctor (name/s, phone number)			
Delegate physician (name/s, phone number)			

Physician's signature:

Date:

Hospice-Hotline: 0810 969 878

This document was developed in cooperation with representatives of the following institutions: palliative unit of the regional medical association; Tyrol Hospice Community; Social center for competence Rum; Emergency Medical Services, Department of anesthesiology and critical care, Innsbruck medical university, Palliative Medicine and Hospice Provision Tyrol, Health Fond Tyrol.

Version: September 2010
Contact: Dr. E.Medicus
E-Mail: Elisabeth.Medicus@hospiz-tirol.at

Table S3. Questionnaire on user satisfaction with the palliative treatment plan.

**Nursing personnel/emergency physician/family doctor**					
Nursing home					
Your feed-back is important and will be considered in an improved version of the palliative treatment plan					
Demographic Data:					
Gender:	☐ female			☐ male	
Age:	<30 years<br>☐		30–50 years<br>☐	>50 years<br>☐	
Does the palliative treatment plan contribute to patient's benefit?	☐ Yes<br>☐ no				
Is there rapid accessibility to the palliative treatment plan as needed?	☐ Yes<br>☐ no				
Where do you retain the palliative treatment plan?					
Does the plan contribute to security in your treatment of patients?	☐ Yes<br>☐ no				
Does the plan influence your communication with patient and relatives?	☐ Yes<br>☐ no				
Do you feel secure in the care of patients since implementation of the plan?	very secure<br>☐<br>1	secure<br>☐<br>2	similar<br>☐<br>3	insecure<br>☐<br>4	very insecure<br>☐<br>5
Does the palliative treatment plan facilitate your work?	facilitate<br>☐<br>1	lighten<br>☐<br>2	similar<br>☐<br>3	hinder<br>☐<br>4	complicate<br>☐<br>5
Are you content with the handling of the palliative treatment plan?	very content<br>☐<br>1	content<br>☐<br>2	similar<br>☐<br>3	discontent<br>☐<br>4	very discontent<br>☐<br>5
How do you judge the efficiency of the palliative treatment plan?	Very efficient<br>☐<br>1	efficient<br>☐<br>2	similar<br>☐<br>3	inefficient<br>☐<br>4	very inefficient<br>☐<br>5

Table S3. *Cont.*

**Questions regarding application of the PTP**					
Which depository for the plan is most suitable?	□ in the health records<br>□ at bedside<br>□ in the nurses quarter<br>□ others:				
How many patients with completed palliative treatment plan did you treat?	0	1–2	3–4	5–6	>6
	□	□	□	□	□
	1	2	3	4	5
How secure do you feel in a conversation dealing with advance care planning?	very secure	secure	similar	insecure	very insecure
	□	□	□	□	□
	1	2	3	4	5
How secure do you feel in the implementation of the palliative treatment plan?	very secure	secure	similar	insecure	very insecure
	□	□	□	□	□
	1	2	3	4	5
Do you require more information for working with the palliative treatment plan?	□ Yes<br>□ no				
If needed, what do you request?					
**Questions regarding application of the PTP**					
Is there need of improvement?<br>➢ Medical background<br>➢ Background of decisions<br>➢ Symptomatic treatment					
Which section of the palliative treatment plan is all right?					
**Questions regarding application of the PTP**					
Is the phrasing clear?	very good	good	satisfying	bad	very bad
	□	□	□	□	□
	1	2	3	4	5
Suggested improvement					
How do you rate information content of the palliative treatment plan?	very good	good	satisfying	bad	very bad
	□	□	□	□	□
	1	2	3	4	5
Suggested improvement					
How do you rate the arrangement of the palliative treatment plan?	very good	good	satisfying	bad	very bad
	□	□	□	□	□
	1	2	3	4	5
Suggested improvement					
How do you rate the clarity of the palliative treatment plan?	very good	good	satisfying	bad	very bad
	□	□	□	□	□
	1	2	3	4	5
Suggested improvement					
Which intervals between up-dates do you recommend?	weekly	monthly	Half a year	yearly	3-year interval
	□	□	□	□	□
	1	2	3	4	5

Table S3. *Cont.*

**Questions regarding application of the PTP**	
Who is participating in advance care planning in your nursing home?	☐ Patient/Resident<br>☐ Physician<br>☐ Nurse<br>☐ Attachment nurse<br>☐ Charge nurse<br>☐ Head of nursing<br>☐ Relative<br>☐ Other: …
Who should participate in advance care planning to your opinion?	
Thank you very much for your specifications!	
